# Transcriptome Analyses of Liver Sinusoidal Endothelial Cells Reveal a Consistent List of Candidate Genes Associated with Endothelial Dysfunction and the Fibrosis Progression

**DOI:** 10.3390/cimb46080473

**Published:** 2024-07-25

**Authors:** Penghui Li, Wenjie Xie, Hongjin Wei, Fan Yang, Yan Chen, Yinxiong Li

**Affiliations:** 1 Center for Health Research, Guangdong Provincial Key Laboratory of Biocomputing, Guangzhou Institutes of Biomedicine and Health, Chinese Academy of Sciences, Guangzhou 510530, China; 2 University of Chinese Academy of Sciences, Beijing 100049, China; 3 Key Laboratory of Stem Cell and Regenerative Medicine, CAS Key Laboratory of Regenerative Biology, Guangzhou Institutes of Biomedicine and Health, Chinese Academy of Sciences, Guangzhou 510530, China; 4 China-New Zealand Joint Laboratory on Biomedicine and Health, State Key Laboratory of Respiratory Disease, Guangzhou 510530, China

**Keywords:** liver fibrosis, liver sinusoidal endothelial cells, endothelial dysfunction, transcriptomics, *SOX4*, *LGALS3*

## Abstract

Liver fibrosis is an important step in the transformation of chronic liver disease into cirrhosis and liver cancer, and structural changes and functional disorders of liver sinusoidal endothelial cells (LSECs) are early events in the occurrence of liver fibrosis. Therefore, it is necessary to identify the key regulatory genes of endothelial dysfunction in the process of liver fibrosis to provide a reference for the diagnosis and treatment of liver fibrosis. In this study, we identified 230 common differentially expressed genes (Co-DEGs) by analyzing transcriptomic data of primary LSECs from three different liver fibrosis mouse models (carbon tetrachloride; choline-deficient, l-amino acid-defined diet; and nonalcoholic steatohepatitis). Enrichment analysis revealed that the Co-DEGs were mainly involved in regulating the inflammatory response, immune response, angiogenesis, formation and degradation of the extracellular matrix, and mediating chemokine-related pathways. A Venn diagram analysis was used to identify 17 key genes related to the progression of liver cirrhosis. Regression analysis using the Lasso–Cox method identified genes related to prognosis among these key genes: *SOX4*, *LGALS3*, *SERPINE2*, *CD52*, and *LPXN*. In mouse models of liver fibrosis (bile duct ligation and carbon tetrachloride), all five key genes were upregulated in fibrotic livers. This study identified key regulatory genes for endothelial dysfunction in liver fibrosis, namely *SOX4*, *LGALS3*, *SERPINE2*, *CD52*, and *LPXN*, which will provide new targets for the development of therapeutic strategies targeting endothelial dysfunction in LSECs and liver fibrosis.

## 1. Introduction

Liver fibrosis is the diffuse overdeposition and abnormal distribution of extracellular matrix in the liver, which is the pathological repair response of the liver to chronic injury and is also a key step in the progression of various chronic liver diseases to cirrhosis and an important link that affects the prognosis of chronic liver diseases. If left untreated, it can progress to cirrhosis and liver cancer and induce various end-stage liver disease complications. Liver fibrosis is caused by the repeated and long-term effects of one or more etiologic factors on the liver, and its main causes include alcohol-associated liver disease, nonalcoholic fatty liver disease, hepatitis B or C infection, autoimmune liver disease, and cholestatic liver injury [1].

In clinicopathologic staging diagnosis, liver fibrosis can be divided into stages I to IV, where IV is the early stage of cirrhosis. And cirrhosis can be subdivided into compensated cirrhosis and decompensated cirrhosis. Cirrhosis is widely prevalent worldwide, with the Global Burden of Disease statistics in 2017 showing that 1.5 billion people worldwide have chronic liver disease or cirrhosis [2]. The number of patients with compensated cirrhosis increased from 65.95 million in 1990 to 112 million in 2017, and the number of patients with decompensated cirrhosis increased from 5.2 million in 1990 to 10.64 million in 2017 [3]. Liver cirrhosis causes a total of 1.32 million deaths worldwide (accounting for 2.4% of total global deaths), two-thirds of which are men [3]. Compared with those in healthy populations, patients with compensated and decompensated cirrhosis have a five-fold and ten-fold increased risk of death, respectively [4]. The 1- and 5-year survival rates for compensated cirrhosis patients were 87.3% and 66.5%, respectively, while the 1- and 5-year survival rates for decompensated cirrhosis patients were 75.0% and 45.4%, respectively [4]. The median survival time for patients with compensated cirrhosis is 12 years, while it decreases to 2–4 years after progression to decompensated cirrhosis [5]. Despite the severity of the current disease situation, there are still no FDA-approved drugs that can effectively treat liver fibrosis or cirrhosis.

The pathogenesis of liver fibrosis is complex and involves the coregulation of multiple cell types, among which endothelial cells, as one of the major components of the hepatic microvascular system, have an important influence on the development of liver fibrosis. Liver sinusoidal endothelial cells (LSECs), the most abundant type of nonparenchymal cell in the liver, have multiple functions, such as blood flow regulation, selective permeability, endocytosis clearance, and immunomodulation, and play important roles in maintaining normal physiological processes and immune homeostasis in the liver [6,7]. LSECs differ from other endothelial cells in that they lack an organized basement membrane and have many fenestrae. These characteristics endow LSECs with selective permeability, facilitating the exchange of oxygen, nutrients, or metabolic waste between liver cells and blood [6,8]. Structural changes in LSECs and their dysfunction are early events in the development of hepatic fibrosis, during which LSECs lose their protective properties and, instead, possess vasoconstrictive, proinflammatory, and prothrombotic functions [9]. In addition, basement membrane formation and loss of fenestrae impede hepatocyte–oxygen exchange, leading to hepatocyte necrosis and apoptosis and the secretion of damage-associated molecular patterns (DAMPs). DAMPs and cytokines secreted by LSECs further activate hepatic stellate cells, leading to excessive production and deposition of extracellular matrix (ECM), thereby promoting the development of liver fibrosis [10]. Therefore, the identification of key genes involved in endothelial dysfunction in LSECs during liver fibrosis may provide new targets for the treatment of liver fibrosis.

Transcriptome analysis, a powerful high-throughput sequencing technology, provides a powerful tool for revealing gene expression profiles and regulatory networks. In this study, we used public datasets from the Gene Expression Omnibus (GEO) database to mine the transcriptome data of primary LSECs in different liver fibrosis model mice, identify common differentially expressed genes (Co-DEGs), and explore the biological functions of these genes to further study the molecular mechanism of endothelial dysfunction in LSECs during liver fibrosis. We then identified genes in the Co-DEGs that were associated with the progression and prognosis of cirrhosis and verified the expression of these genes in liver fibrosis model mice. This study, focusing on LSECs and the molecular bases of endothelial dysfunction during liver fibrosis from different mice models, found that *SOX4*, *LGALS3*, *SERPINE2*, *CD52*, and *LPXN* are potential key genes associated with endothelial dysfunction in liver fibrosis.

## 2. Materials and Methods

### 2.1. Data Sources for the Microarray

Gene expression profiling datasets were downloaded from the GEO (http://www.ncbi.nlm.nig.gov/geo/, accessed on 20 March 2024) database (Table 1), including transcriptome data of primary LSECs induced by 3 different models of hepatic fibrosis in mice, namely carbon tetrachloride (CCL4) (GSE120281), choline-deficient, l-amino acid-defined (CDAA) diet (GSE140994), and nonalcoholic steatohepatitis (NASH) (GSE119340). The GSE84044 and GSE139602 datasets were used for different stages of human cirrhosis, and GSE14520 was used for the prognosis of patients with HBV-associated cirrhosis and hepatocellular carcinoma.

### 2.2. Identification of Differentially Expressed Genes

Differential analysis of the transcriptome datasets of LSECs induced by three different models of liver fibrosis in mice was performed using the R package “limma”, and volcano maps and heatmaps were generated using the online platform hiplot (https://hiplot.org, accessed on 25 March 2024) [17]. The screening criteria were a |log2-fold change (FC)|  >  1 and an adjusted *p*-value  <  0.05. On this basis, the above three groups of differentially expressed genes and genes related to endothelial dysfunction of liver fibrosis-derived LSECs were clustered, and a Venn diagram was drawn to visualize a collection of differentially expressed genes related to liver fibrosis dysfunction in LSECs.

### 2.3. Functional Enrichment and GSEA

To gain a deeper understanding of the main biological functions of the Co-DEGs, we used the Metascape (http://metascape.org, accessed on 25 March 2024) platform to analyze the Gene Ontology (GO), Kyoto Encyclopedia of Genes and Genomes (KEGG), and Reactome pathways [18]. An adjusted *p*-value < 0.05 was considered significant. Moreover, to gain a deeper understanding of the biological processes associated with endothelial dysfunction in LSECs during liver fibrosis, we performed GSEA on the GSE120281 dataset using the clusterProfiler (version 4.8.1) software package.

### 2.4. Selection and Analysis of PPI Network Core DEGs

PPI networks for Co-DEGs were constructed using STRING (http://string-db.org, accessed on 10 April 2024) (version 12.0). Combinatorial scores above 0.4 were used as a selection threshold. The PPI networks were then visualized using Cytoscape (http://www.cytoscape.org, accessed on 10 April 2024) (version 3.10.2). Six common algorithms (EPC, MCC, MNC, Radiality, Degree, and Closeness) of the Cytoscape plugin cytoHubba were used to evaluate and filter the core Co-DEGs [19].

### 2.5. Machine Learning of Lasso and Random Forest

We used the Sangerbox (http://vip.sangerbox.com/, accessed on 12 April 2024) platform to integrate survival time, survival status, and gene expression data and used the Lasso–Cox method to perform regression analysis [20]. In addition, univariate logistic regression was performed on the key genes.

### 2.6. Liver Fibrosis Mouse Model

Mouse liver fibrosis models were generated using BDL and carbon tetrachloride (CCL4). Six-week-old C57BL/6 male mice were subjected to BDL for 18 days after BDL, while control mice had their wounds sutured after laparotomy. The CCL4 liver fibrosis model was induced by intraperitoneal injection of 1 µL/g body weight CCL4 (Sigma-Aldrich #3319961, Saint Louis, MO, USA) into 6-week-old C57BL/6 male mice. The control mice were injected with the same dose of olive oil twice a week for 6 weeks. Four mice were used for each of the above groups. No more than 5 mice were kept in each cage. All operations were performed in accordance with the “GIBH Experimental Animal Breeding, Management and Use Manual” and reported to the Experimental Animal Center of Guangzhou Institutes of Biomedicine and Health, Chinese Academy of Sciences.

### 2.7. Histology and Immunohistochemistry

Immediately after sacrifice, mouse livers were fixed with 4% paraformaldehyde, and after tissue processing and paraffin embedding, 4 μm-thick sections were cut for further staining. After deparaffinization, the paraffin sections were stained with H&E or Sirius red, followed by dehydration and mounting. For immunohistochemistry, after deparaffinization of the sections, heat-induced antigen retrieval was conducted using citrate-EDTA buffer for 15 min, followed by blocking at room temperature for 1 h. Subsequently, the sections were incubated overnight with a primary antibody against α-SMA (abcam, Cambridge, UK, ab124964, 1:1000) or LYVE1 (abcam, ab281587, 1:1000) and with an HRP-labeled goat anti-rabbit antibody (KC-RB-035; Aksomics, Shanghai, China) utilized as the secondary antibody. Dako AEC substrate chromogen (K3468, Agilent Technologies, Santa Clara, CA, USA) was added for 1 min, and counterstaining with hematoxylin was performed to stain the nuclei, followed by dehydration and mounting.

### 2.8. Quantitative Reverse Transcription-PCR (qRT-PCR)

RNA was extracted using TRIzol (15596-018, Life Technologies, Carlsbad, CA, USA) and reverse transcribed to cDNA using ReverTraAce qPCR RT Master Mix (FSQ-301, Toyobo, Tokyo, Japan). cDNA was subsequently subjected to qRT-PCR using ChamQ SYBR qPCR Master Mix (Vazyme, Nanjing, China, Q311-02). Using *18s* as an endogenous control, the results were calculated using the 2^−ΔΔCt^ method. The sequences of primers used are listed in Table 2.

### 2.9. Statistical Analysis

Differences between two groups were compared using an unpaired Student’s *t* test. The correlation between two groups was calculated using Pearson’s correlation analysis. The data are presented as the means ± SDs. A *p*-value less than 0.05 indicated statistical significance.

## 3. Results

### 3.1. Identification of Co-DEGs Associated with Endothelial Dysfunction in Liver Fibrosis LSECs

**Figure 1:**
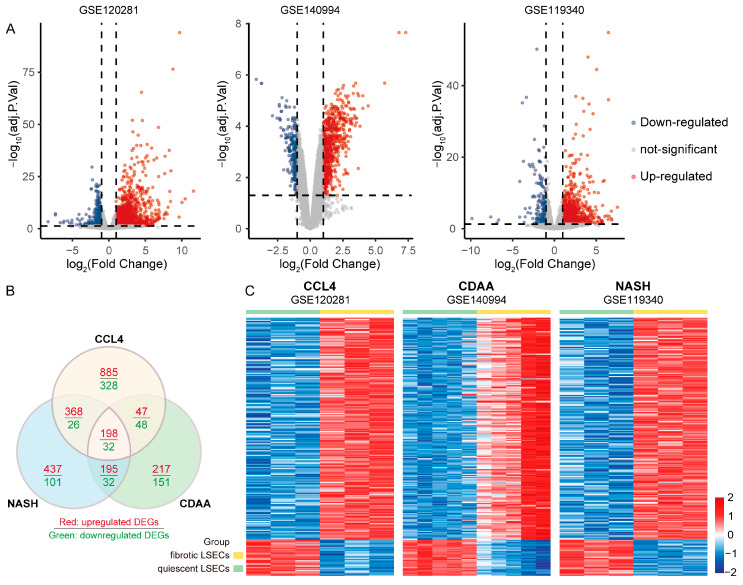
Identification of Co-DEGs in LSECs from mice with liver fibrosis (CCL4, CDAA, and NASH). (**A**) Volcano plot showing DEGs in the GSE120281, GSE140994, and GSE119340 datasets. (**B**) Venn diagram showing Co-DEGs in LSECs from different liver fibrosis models. (**C**) Heatmap showing the expression of Co-DEGs in the dataset.

To identify differentially expressed overlapping dysfunction-related genes in liver fibrosis-derived LSECs, we first performed differential expression analysis of liver fibrosis LSECs induced by different models. The results showed that 1498 upregulated and 434 downregulated genes were differentially expressed in LSECs during liver fibrosis induced by CCL4 in the GSE120281 dataset (Figure 1A). In the GSE140994 dataset, 657 upregulated genes and 263 downregulated genes were differentially expressed in LSECs associated with liver fibrosis induced by CDAA diet (Figure 1A). A total of 1198 genes, whose expression was differentially expressed in LSECs from mice with diet-induced NASH-related liver fibrosis, were upregulated, and 191 genes were downregulated (Figure 1A). Supplementary Table S1 provides detailed information on the above differentially expressed genes. Cluster analysis of the above differentially expressed genes revealed a total of 230 Co-DEGs, including 198 upregulated genes and 32 downregulated genes (Figure 1B and Supplementary Table S2). A heatmap of the expression of these Co-DEGs in the dataset of LSECs from mice with liver fibrosis induced by different models is shown in Figure 1C.

### 3.2. Functional Enrichment and GSEA of Co-DEGs

**Figure 2:**
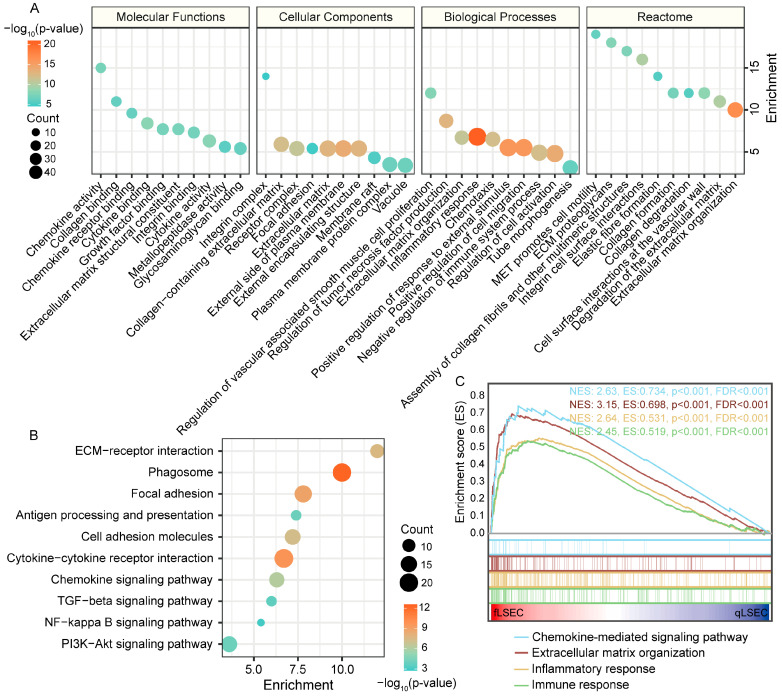
Functional enrichment analysis of Co-DEGs. (**A**) GO and Reactome functional annotation. (**B**) KEGG pathway enrichment analysis. (**C**) GSEA validation of selected signaling pathways in the GSE120281 dataset.

To investigate the role of Co-DEGs in the endothelial dysfunction process of liver fibrotic LSECs, GO, Reactome, and KEGG functional enrichment analyses were conducted. GO enrichment analysis revealed that the main molecular functions of these genes included chemokine activity, collagen binding, chemokine receptor binding, cytokine binding, extracellular matrix structural constituents, growth factor binding, integrin binding, cytokine activity, metallopeptidase activity, and glycosaminoglycan binding (Figure 2A). The functions of Co-DEGs in cellular components included integrin complex, collagen-containing extracellular matrix, external side of the plasma membrane, extracellular matrix, external encapsulating structure, receptor complex, focal adhesion, membrane raft, plasma membrane protein complex, and vacuole. At the same time, the biological processes in which the Co-DEGs participate mainly included the regulation of vascular-associated smooth muscle cell proliferation, the regulation of tumor necrosis factor production, inflammatory response, extracellular matrix organization, chemotaxis, positive regulation of cell migration, positive regulation of response to external stimulus, negative regulation of immune system process, regulation of cell activation, and tube morphogenesis (Figure 2A). Reactome pathway analysis revealed that Co-DEGs are involved mainly in the formation and degradation of the extracellular matrix and collagen (Figure 2A).

KEGG pathway analysis revealed that the signaling pathways associated with the Co-DEGs included ECM–receptor interaction, phagosome, focal adhesion, antigen processing and presentation, cell adhesion molecules, cytokine–cytokine receptor interaction, chemokine signaling pathways, TGF-beta signaling pathway, NF-kappa B signaling pathway, and PI3K-Akt signaling pathway (Figure 2B). The above functional enrichment analysis revealed that Co-DEGs from LSECs during liver fibrosis mainly regulate inflammation, immunity, extracellular matrix formation, and degradation and mediate chemokine-related pathways. Furthermore, we performed gene set enrichment analysis (GSEA) on these major pathways in the CCL4-induced liver fibrosis dataset GSE120281. The results showed that the chemokine-mediated signaling pathway, extracellular matrix organization, inflammatory response, and immune response were activated (Figure 2C).

### 3.3. Screening of the Hub Genes via the Protein–Protein Interaction Network

**Figure 3:**
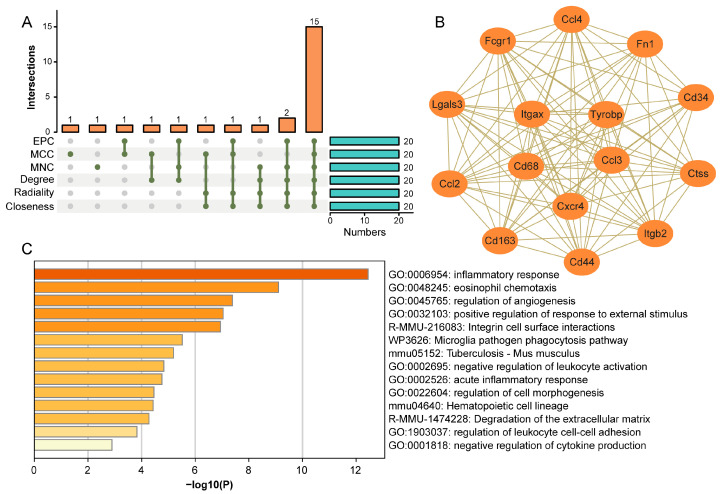
Screening and functional analysis of the hub genes for the Co-DEGs. (**A**) Six algorithms for screening overlapping regulatory genes. (**B**) PPI network of the overlapping regulatory genes. (**C**) Functional enrichment analysis of overlapping regulatory genes.

In order to explore the hub genes in these 230 Co-DEGs and their related biological functions, by using the STRING analyses, protein–protein interaction (PPI) of these 230 Co-DEGs was obtained, followed with cytoHubba/Cytoscape and six algorithms analyses, and the 20 hub genes were generated (Table 3). The 15 overlapping hub genes were identified via Venn diagram analysis (Figure 3A). These hub genes included numerous chemokines and receptors (*Ccl2*, *Ccl3*, *Ccl4*, and *Cxcr4*) and form a complex interaction network (Figure 3B). Enrichment analysis revealed that these genes are involved in regulating biological processes, including the inflammatory response, eosinophil chemotaxis, angiogenesis regulation, integrin cell surface interactions, ECM, and cytokine production (Figure 3C).

### 3.4. Correlations of Co-DEGs with the Progression of Human Liver Cirrhosis

**Figure 4:**
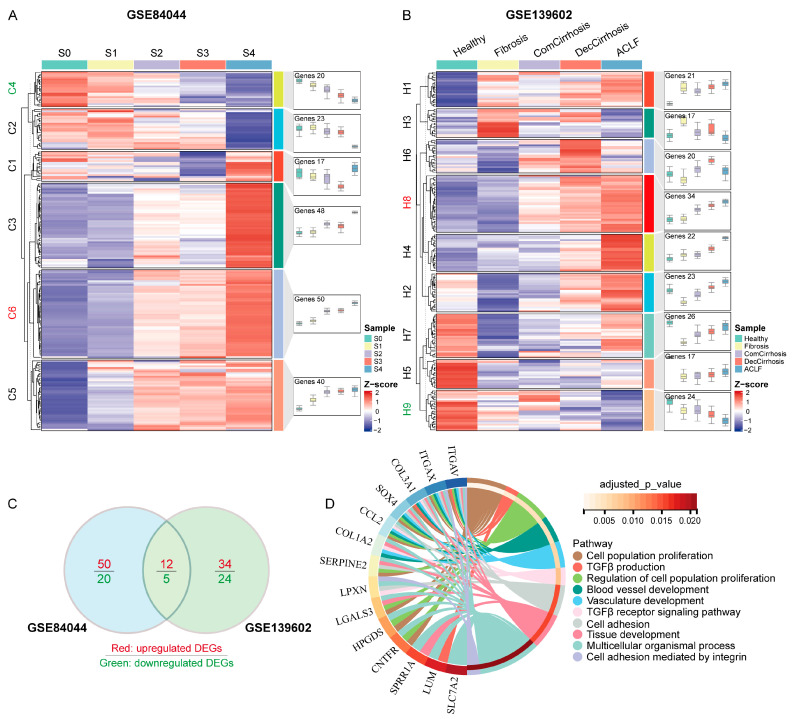
Pseudotemporal analysis of Co-DEGs involved in the progression of human cirrhosis. (**A**) Expression clustering of Co-DEGs in different grades of liver fibrosis. (**B**) Expression clustering of Co-DEGs in different stages of liver cirrhosis. (**C**) Common key genes associated with the progression of liver cirrhosis among the Co-DEGs. (**D**) Functional enrichment analysis of key genes.

To further screen the genes among the Co-DEGs correlated with the progression of cirrhosis, we performed pseudotemporal analysis of the expression of these genes at different stages of liver fibrosis. GSE84044 contains data on 124 patients with liver fibrosis grades ranging from S0 to S4. The expression profiles of Co-DEGs in GSE84044 were extracted, and the mean value was taken for each set of data. Trend analysis was performed, and an expression heatmap was generated. The results showed that the expression of Co-DEGs in the GSE84044 dataset could be categorized into six groups (Figure 4A), which included a continuously upregulated C6 cluster (50 genes) and a continuously downregulated C4 cluster (20 genes). The GSE139602 dataset contains six groups: healthy controls, patients with fibrosis, patients with compensated cirrhosis, patients with decompensated cirrhosis, and patients with acute-on-chronic liver failure (ACLF). The expression of Co-DEGs in the GSE139602 dataset can be categorized into nine classes (Figure 4B), including the continuously upregulated H8 cluster (34 genes) and the continuously downregulated H9 cluster (24 genes). Venn diagram analysis of the above differentially expressed genes revealed twelve genes whose expression was serially upregulated and five genes whose expression was serially downregulated during the progression of liver fibrosis (Figure 4C). The biological processes associated with these genes included mainly cell population proliferation, transforming growth factor beta production, regulation of cell population proliferation, blood vessel development, vasculature development, the transforming growth factor beta receptor signaling pathway, cell adhesion, tissue development, multicellular organismal processes, and cell adhesion mediated by integrins (Figure 4D). Among them, *ITGAV*, *ITGAX*, *SOX4*, *CCL2*, *SERPINE2*, *LPXN*, *LGALS3*, *HPGDS*, and *CNTFR* can regulate multiple biological processes, while *MPP6*, *CD52*, and *SLC16A10* are not involved in the above biological processes.

### 3.5. Co-DEGs and Survival Status of Patients with Liver Cirrhosis

**Figure 5:**
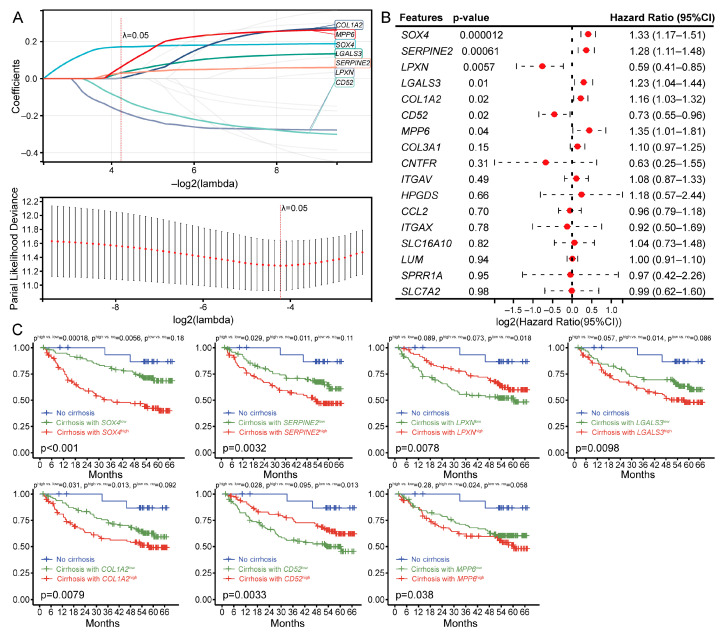
Machine analysis and prognostic results of key genes in the HBV-associated cirrhosis hepatocellular carcinoma patient dataset GSE14520. (**A**) Lasso analysis of key genes in GSE14520. (**B**) Univariate logistic regression analysis of key genes in GSE14520. (**C**) Prognostic analysis of key genes in cirrhotic HCC patients; *p*-values for cluster-pair comparisons at the top and multivariate *p*-values at the bottom.

Due to the small proportion of biopsies from cirrhotic patients, it is difficult to obtain a large sample size for transcriptome analysis and prognostic tracking; therefore, we used the GSE14520 hepatocellular carcinoma patient transcriptome dataset. This dataset contains transcriptomic data and prognostic information on 386 patients with resected hepatocellular carcinoma, and after excluding non-HBV-positive patients and some missing data, a total of 212 samples were included in the present analysis, 195 of which had comorbid cirrhosis. We performed Lasso regression on the above 17 Co-DEGs that correlated with cirrhosis progression in the GSE14520 dataset to further screen for characterized genes associated with prognosis. Lasso regression is a machine-learning algorithm involving the assumption of a linear relationship and an L1 regularization penalty. First, Lasso regression with the minimized binomial deviation was performed through 10-fold cross-validation. Then, genes with nonzero regression coefficients were selected for the feature genes of the Co-DEGs. Seven Co-DEGs (*COL1A2*, *MPP6*, *SOX4*, *LGALS3*, *SERPINE2*, *LPXN*, and *CD52*) were included in the simplified Lasso regularization model from GSE14520 (Figure 5A). Moreover, the prognostic significance of each gene was assessed by integrating survival time, survival status, and gene expression data using univariate logistic regression analysis (Figure 5B). The results showed that the above genes, *SOX4*, *SERPINE2*, *LPXN*, *LGALS3*, *COL1A2*, *CD52*, and *MPP6*, which were screened by Lasso regression, were correlated with prognosis. Furthermore, we grouped all patients according to noncirrhotic and cirrhotic low expression and cirrhotic high expression and generated survival curves for the seven key genes (Figure 5C). The log-rank test was conducted to analyze the difference of survival curves among those three clusters of the seven genes, and the *p*-values for each cluster pair were listed in Figure 5C. Higher expressions of *SOX4*, *SERPINE2*, *LGALS3*, and *COL1A2* were associated with poor prognosis; however, the lower expressions of *LPXN* and *CD52* were also associated with poor prognosis, but either higher or lower expressions of *MPP6* had no significant association with prognosis. In more detail, the *p*-values of the survival curves with high expression vs. the counterpart of low expression are as follows: *SOX4* (*p*^high vs. low^ = 0.00018), *SERPINE2* (*p*^high vs. low^ = 0.029), *CD52* (*p*^high vs. low^ = 0.028), and *COL1A2* (*p*^high vs. low^ = 0.031). The *p*-values of *LGALS3*^high vs. low^ and *LPXN*^high vs. low^ were close to 0.05; however, *MPP6*^high vs. low^ reached a *p*-value of 0.28 (Figure 5C).

**Figure 6:**
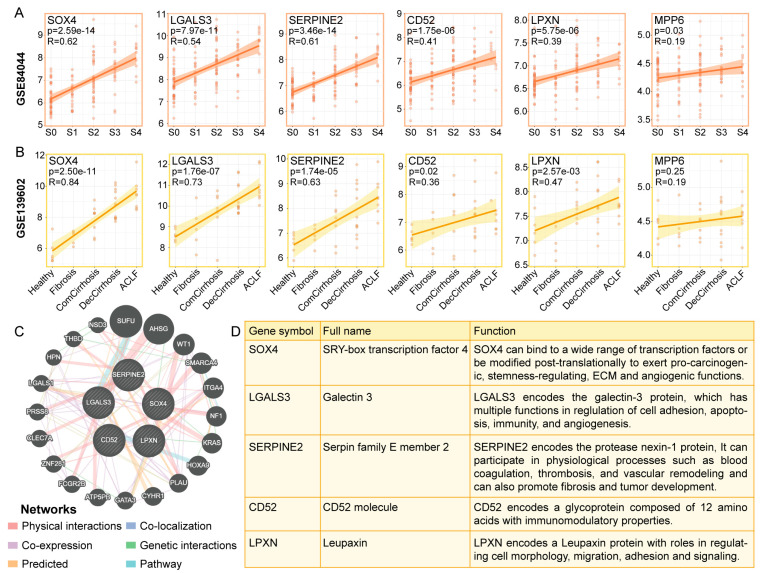
Correlation and interaction analysis of key genes associated with liver fibrosis and liver disease progression. (**A**) Correlations between *SOX4*, *LGALS3*, *SERPINE2*, *CD52*, *LPXN*, and *MPP6* expression and liver fibrosis grade. (**B**) Correlations of *SOX4*, *LGALS3*, *SERPINE2*, *CD52*, *LPXN*, and MPP6 expression and liver disease progression. (**C**) GeneMANIA was used to analyze the coexpression network and related functions of key genes. (**D**) Full name and function of key genes.

Considering that COL1A2 is a collagen, we performed a correlation analysis between *SOX4*, *LGALS3*, *SERPINE2*, *CD52*, *LPXN*, and *MPP6* and the progression of cirrhosis. The Pearson correlation coefficient was |R| > 0.3, and a *p*-value < 0.05 indicated a correlation. *SOX4*, *LGALS3*, and *SERPINE2* were strongly correlated with liver fibrosis grade and liver disease progression, with correlation coefficients (R) as high as 0.62 and 0.84 for *SOX4* and 0.54 and 0.73 for *LGALS3*, respectively (Figure 6A,B). The correlation coefficients (R values) of *SERPINE2* with liver fibrosis grade and liver disease progression were 0.61 and 0.63, respectively. *CD52* and *LPXN* were moderately correlated with both liver fibrosis grade and liver disease progression, while *MPP6* was weakly correlated with liver fibrosis grade and liver disease progression (Figure 6A,B). However, the prognostic value of *MPP6* did not differ between the high- and low-expression groups with cirrhosis (Figure 5C). Therefore, we analyzed the coexpression network and related functions of five key genes, *SOX4*, *LGALS3*, *SERPINE2*, *CD52*, and *LPXN*, using GeneMANIA. These genes exhibited a complex PPI network with 77.64% physical interactions, 8.01% coexpression, 5.37% predicted interactions, 3.63% colocalization, 2.87% genetic interactions, and 1.88% pathways (Figure 6C). Figure 6D shows the full names and related functions of these key genes.

### 3.6. Verification of the Expression of Key DEGs in Mice with Liver Fibrosis

**Figure 7:**
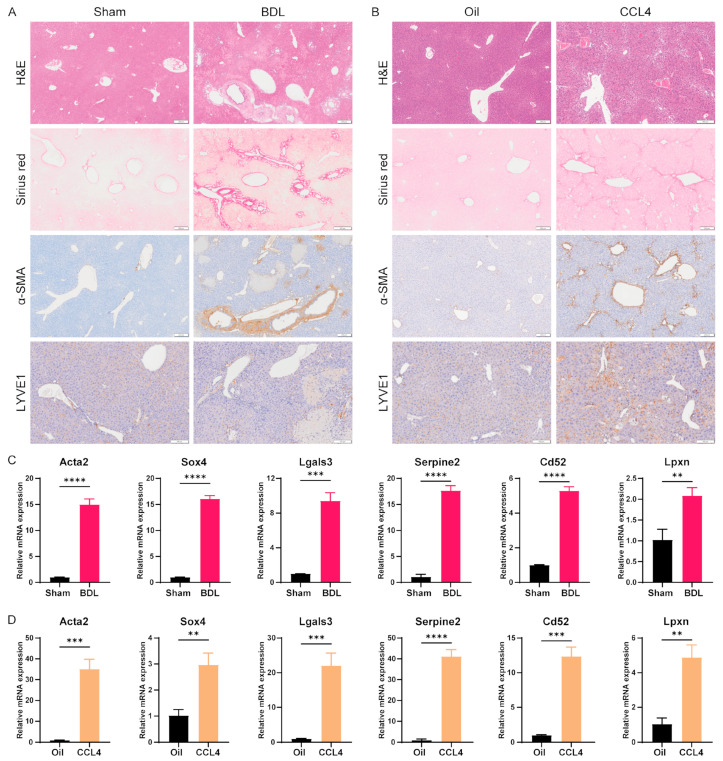
Verification of the expression of key genes in mice with BDL- and CCL4-induced liver fibrosis. (**A**) H&E, Sirius red, α-SMA, and LYVE1 staining of BDL model mice. (**B**) H&E, Sirius red, α-SMA, and LYVE1 staining of CCL4 model mice. (**C**) qRT-PCR was used to detect the expression of *Acta2*, *Sox4*, *Lgals3*, *Sepine2*, *Cd52*, and *LPXN* in the liver tissue of BDL model mice. (**D**) qRT-PCR was used to detect the expression of *Acta2*, *Sox4*, *Lgals3*, *Sepine2*, *Cd52*, and *Lpxn* in the liver tissue of CCL4 model mice. The data are presented as the means ± SDs. ** *p* < 0.01, *** *p* < 0.001, **** *p* < 0.0001. Scale bar: 200 μm (H&E, Sirius red, and α-SMA); 100 μm (LYVE1).

To verify the expression of the above key DEGs in the livers of mice with fibrosis, we generated bile duct ligation (BDL) and CCL4 liver fibrosis models in C57BL/6 mice. Hematoxylin–eosin (H&E) staining revealed that the hepatocytes in the BDL and CCL4 groups were disordered, the liver lobule structure was blurred, the liver sinusoids were expanded, and obvious fibrotic areas were apparent (Figure 7A,B). Sirius red is a strongly acidic dye that binds to collagen fibers and appears red in color. Compared with those in the control group, intrahepatic collagen deposition was greater in the BDL and CCL4 model groups (Figure 7A,B).

Immunohistochemical staining revealed obvious α-SMA-positive staining in hepatic stellate cells around liver sinusoids and in fibrotic areas, indicating that these cells had been activated and transformed into fibrosis-related fibroblasts. Immunohistochemical staining for LYVE1, a marker of LSECs, showed that LYVE1 expression was downregulated during liver fibrosis and was accompanied by disorganization of LSECs (Figure 7A,B). In addition, quantitative reverse transcription-PCR (qRT-PCR) was performed on liver tissue samples, and the RNA levels of Acta2 were significantly increased in the BDL and CCL4 groups (Figure 7C,D). These results indicate that the liver fibrosis model was successfully established. Subsequently, we conducted qRT-PCR analysis to examine the expression of five key genes (*SOX4*, *LGALS3*, *SERPINE2*, *CD52*, and *LPXN*) in the liver tissues of mice with liver fibrosis. The results showed a significant increase in the expression of all key genes during liver fibrosis (Figure 7C,D). Among these genes, *SOX4*, *LGALS3*, *SERPINE2*, and *CD52* were upregulated more strongly in fibrotic livers, while *LPXN* was upregulated less strongly. Therefore, *SOX4*, *LGALS3*, *SERPINE2*, *CD52*, and *LPXN* may serve as key regulatory genes for endothelial dysfunction in liver fibrosis LSECs.

## 4. Discussion

Liver fibrosis is an important stage in the transformation of chronic liver disease to cirrhosis and hepatocellular carcinoma and is mainly characterized by the massive production of ECM and its deposition in the sinusoidal space, leading to structural destruction of the hepatic lobules and revascularization. The pathological process underlying the occurrence and development of liver fibrosis is very complex and involves liver parenchymal cells, nonparenchymal cells, a variety of cytokines, and noncellular components. Among these, hepatic microenvironmental changes caused by dysfunction of LSECs are the initial events in the development of hepatic fibrosis and an important part of disease progression. The hepatic sinusoids are the main site of substance exchange between hepatocytes and blood, and LSECs play an important role as a major component of the hepatic sinusoids. LSECs play a crucial role in mediating complex physiological functions, such as energy and material exchange, phagocytosis, and immune regulation, between hepatic sinusoidal blood flow and hepatic parenchymal cells, utilizing selective permeability of the cell membrane, expression of various endocytic clearance receptors, and secretion of cytokines and inflammatory mediators [21,22,23]. Under physiological conditions, LSECs have antifibrotic and anti-inflammatory properties, but dysfunctional LSECs can induce pathological vascular proliferation and collagen deposition in liver sinusoids, promote liver tissue inflammation, and lead to the progression of liver fibrosis [24]. Therefore, maintaining or restoring the healthy phenotype of LSECs is an important strategy for preventing or alleviating liver diseases.

Our study identified 230 Co-DEGs by analyzing transcriptomic data of primary LSECs from three different liver fibrosis mouse models (CCL4, CDAA, and NASH). These Co-DEGs are mainly involved in regulating inflammation, immunity, extracellular matrix formation, and degradation and mediating chemokine-related pathways. The Co-DEGs included numerous upregulated chemokines or their receptors (*Ccl2*, *Cxcl5*, *Cxcr4*, etc.) (Supplementary Tables S1 and S2), which signifies that LSECs have the ability to activate inflammatory cells to participate in the inflammatory response of hepatic tissues and to activate hepatic stellate cells and induce the secretion of the latter into the ECM [25,26,27,28]. Moreover, GO enrichment analysis of the Co-DEGs and hub genes also revealed that LSECs participate in angiogenesis during the occurrence of liver fibrosis. The terminal differentiation marker *Cd209b* of LSECs was significantly downregulated in all three groups of liver fibrosis models [29], whereas the capillarization marker gene *Cd34* and the Edn1 receptor *Ednrb* of LSECs were significantly upregulated in all three groups of liver fibrosis models (Supplementary Table S1) [30,31]. Fibrinogen, collagen, nidogen, and laminins are basement membrane components of endothelial cells [32], and genes corresponding to the expression of these proteins are all significantly upregulated during liver fibrosis (Supplementary Table S1), which is consistent with the synthesis of numerous ECM and collagen proteins according to enrichment analysis (Figure 2).

By analyzing the Co-DEGs associated with the progression and prognosis of liver cirrhosis, we identified five key genes: *SOX4*, *LGALS3*, *SERPINE2*, *CD52*, and *LPXN*. These genes are all gradually upregulated during the disease progression of patients with liver cirrhosis. Additionally, we verified the upregulation of these genes in liver fibrosis in the BDL- and CCL4-induced mouse models. Interestingly, among these key genes, *LGALS3* is the hub gene in the PPI interaction network and is the only key gene that has been reported to regulate cirrhosis progression. *LGALS3* encodes the galectin-3 protein, which has multiple functions, including regulation of cell adhesion, apoptosis, immunity, and angiogenesis. Its expression is upregulated in numerous fibrotic diseases, such as lung, myocardial, and renal diseases [33,34,35]. Galectin-3 is also upregulated in patients with cirrhosis and hepatocellular carcinoma, and its high expression correlates with immune infiltration, invasion, metastasis, and poor prognosis in hepatocellular carcinoma patients [36,37,38,39]. In liver fibrosis, galectin-3 is required for TGF-β-mediated fibroblast activation and ECM production [40]. Currently, several inhibitors targeting Galectin-3 have been developed for disease therapy [41,42,43]. Clinical trials of the galectin-3 inhibitors GB1211 and Belapectin for the treatment of liver cirrhosis are currently in phase II clinical trials (ClinicalTrials.gov Identifier: NCT05009680, NCT02462967). The galectin-3 inhibitor GB0139, which targets idiopathic pulmonary fibrosis, is also in clinical phase IIb (ClinicTrials.gov Identifier: NCT03832946).

Based on our analyses on a publicly available transcriptome database, we found that *SOX4* was upregulated in LSECs from different liver fibrosis models. *SOX4* is a member of the SOX transcription factor family and plays an important role in cell fate determination and tissue morphogenesis [44,45]. *SOX4* expression is not only associated with biliary reprogramming and steatohepatitis but also with hepatocellular carcinoma progression [46]. During liver development, *SOX4* is involved in the development of primary cilia and in the normal formation, elongation, and branching of the biliary tree [47]. whereas cholestatic liver injury can upregulate the expression of *SOX4* and induce changes in the chromatin structure of hepatocytes, thereby promoting biliary reprogramming [48]. *SOX4* in the liver binds to the proximal promoter region of SREBP-1c and upregulates SREBP-1c expression, promoting hepatic steatosis [49]. High expression of the proto-oncogene *SOX4* in hepatocellular carcinoma tends to be associated with increased epithelial mesenchymal transformation, proliferation, metastasis, multidrug resistance, and decreased apoptosis and is correlated with a poorer prognosis in patients with hepatocellular carcinoma [50,51,52]. However, until now, there has been no report indicating a linkage of *SOX4* with liver fibrosis. Whether the upregulated expression of SOX4 in liver fibrosis LSECs is related to LSEC capillarization or the disappearance of fenestrae and the formation of basement membrane as well as its specific regulatory mechanisms still need further investigations.

*SERPINE2* encodes the protease nexin-1 protein, a member of the serine protease inhibitor (SERPIN) superfamily, which is synthesized mainly by endothelial cells, smooth muscle cells, and fibroblasts, among others [53]. It can participate in physiological processes, such as blood coagulation, thrombosis, and vascular remodeling, and can also promote fibrosis and tumor development [53,54,55]. *SERPINE2* inhibits EGFR protein degradation through c-Cbl-mediated ubiquitination in hepatocellular carcinoma and activates the EGFR signaling pathway to promote hepatocellular carcinoma metastasis, whereas inhibition of the SERPINE2–EGFR axis both combats distant metastasis and sensitizes cells to therapeutic agents such as sorafenib [56]. *CD52* is a glycoprotein composed of 12 amino acids and anchored to the cell membrane by glycosylphosphatidylinositol, which is predominantly expressed in lymphocytes and monocytes. The main role of *CD52* is to regulate immune responses, especially by regulating the activation and proliferation of lymphocytes to regulate the degree and nature of immune cell-mediated immune responses [57,58]. Alemtuzumab, a polyclonal antibody targeting *CD52* on T and B cells, has been approved by the FDA for the treatment of leukemia and multiple sclerosis. Alemtuzumab selectively binds to *CD52* molecules on the surface of B cells and T cells, inhibits abnormal activation of lymphocytes, and, thereby, delays the occurrence of multiple sclerosis [59]. Other than the discovery that *CD52* is a key gene for angiogenesis in endothelial cells in Hashimoto’s thyroiditis [60], no additional in-depth studies involving the mechanism of CD52-regulated angiogenesis have been conducted, and the role of CD52-regulated angiogenesis in hepatic fibrosis needs to be further explored. *LPXN* encodes the leupaxin protein, which functions mainly in the extracellular matrix–intracellular junction structure and has roles in regulating cell morphology, migration, adhesion, and signaling [61,62,63].

## 5. Conclusions

In our study, we identified endothelial dysfunction in LSECs during liver fibrosis using bioinformatics analysis and found that the dysfunction mainly involves the inflammatory response, immune response, angiogenesis, the formation and degradation of the extracellular matrix, and the mediation of chemokine-related pathways. Moreover, we identified five key regulatory genes for endothelial dysfunction in liver fibrosis LSECs: *SOX4*, *LGALS3*, *SERPINE2*, *CD52*, and *LPXN*. We verified that the expression of these genes was upregulated in a mouse model of liver fibrosis. This study not only enhances our understanding of endothelial dysfunction during liver fibrosis but also provides potential biomarkers and therapeutic targets for the prevention, diagnosis, and treatment of liver fibrosis.

## Figures and Tables

**Table 1:** Basic information about the GEO datasets.

Dataset	Platform	Etiology	Number of Samples	Ref.
GSE120281	GPL21493	CCL4 liver fibrosis mice	Fibrotic LSECs = 3 Quiescent LSECs = 3	[11]
GSE140994	GPL24557	CDAA liver fibrosis mice	Fibrotic LSECs = 5 Quiescent LSECs = 5	[12]
GSE119340	GPL23479	NASH liver fibrosis mice	Fibrotic LSECs = 3 Quiescent LSECs = 3	[13]
GSE84044	GPL570	Human HBV cirrhosis patients	S0 = 43 S1 = 20 S2 = 33 S3 = 18 S4 = 10	[14]
GSE139602	GPL13667	Human chronic liver disease patients	Healthy = 6 Fibrosis = 5 Compensated cirrhosis = 8 Decompensated cirrhosis = 12 ACLF = 8	[15]
GSE14520	GPL3921	Human hepatocellular carcinoma patients	HCC = 212	[16]

**Table 2:** Primer sequences for qRT-PCR.

Gene	Forward (5′ > 3′)	Reverse (5′ > 3′)
*Sox4*	CCTCGCTCTCCTCGTCCT	TCGTCTTCGAACTCGTCGT
*Lgals3*	CACTGACGGTGCCCTATGAC	TTGGGTTTCACTGTGCCCAT
*Serpine2*	CAGATCATCAAGTCACGGCCT	ACCGTGGAGAGCTGCTTCTTT
*Lpxn*	GCTGCTCCCATCACAGATAAAGTG	TCGGCAGTATGGCTTCTTGTCCTTC
*Cd52*	CTCTTCCTCACTATCATTCTTCTGG	CTTTAGCCTCCTTGGATATCTGCTA
*Acta2*	TCGGATACTTCAGCGTCAGGA	GTCCCAGACATCAGGGAGTAA
*18s*	GTCTGTGATGCCCTTAGATG	AGCTTATGACCCGCACTTAC

**Table 3:** The top 20 Co-DEGs rank in cytoHubba.

EPC	MCC	MNC	Degree	Radiality	Closeness
Itgax	Itgax	Mmp13	Itgax	Itgax	Itgax
Igf1	Cybb	Itgax	Igf1	Cybb	Cybb
Cd68	Cd68	Igf1	Cd68	Igf1	Igf1
Cxcr4	Cd74	Cd68	Cd74	Cd68	Cd68
Ctss	Cxcr4	Cxcr4	Cxcr4	Cxcr4	Cxcr4
Tyrobp	Ctss	Ctss	Ctss	Ctss	Ctss
Ccl4	Tyrobp	Tyrobp	Tyrobp	Tyrobp	Tyrobp
Cd14	Ccl4	Ccl4	Ccl4	Ccl4	Ccl4
Cd44	Cd14	Cd44	Cd44	Cd44	Cd44
Ccl2	Cd44	Ccl2	Ccl2	Ccl2	Ccl2
Cx3cr1	Ccl2	Fn1	Fn1	Cx3cr1	Cx3cr1
Fn1	Cx3cr1	Cd34	Cd34	Fn1	Fn1
Cd34	Fn1	Lgals3	Lgals3	Cd34	Cd34
Lgals3	Cd34	Cd163	Cd163	Lgals3	Lgals3
Cd163	Trem2	Src	Src	Cd163	Cd163
Ccl3	Lgals3	Ccl3	Ccl3	Src	Src
Timp1	Cd163	Timp1	Timp1	Ccl3	Ccl3
Itgb2	Ccl3	Itgb2	Itgb2	Timp1	Timp1
Col1a1	Itgb2	Col1a1	Col1a1	Itgb2	Itgb2
Fcgr1	Fcgr1	Fcgr1	Fcgr1	Fcgr1	Fcgr1

EPC, edge percolated component; MCC, maximum clique centrality; MNC, maximum neighborhood component.

## Data Availability

The raw data of this study are derived from the GEO data portal (https://www.ncbi.nlm.nih.gov/geo/, accessed on 20 March 2024), which are publicly available databases.

## Supplementary Materials

The following supporting information can be downloaded at: https://www.mdpi.com/article/10.3390/cimb46080473/s1, Table S1: The details of the DEGs; Table S2: The overlapping differential genes.
